# Autonomic breathing abnormalities in Rett syndrome: caregiver perspectives in an international database study

**DOI:** 10.1186/s11689-017-9196-7

**Published:** 2017-04-28

**Authors:** Jessica Mackay, Jenny Downs, Kingsley Wong, Jane Heyworth, Amy Epstein, Helen Leonard

**Affiliations:** 1 Telethon Kids Institute, The University of Western Australia, PO Box 855, West Perth, WA 6872 Australia; 2 School of Population Health, The University of Western Australia, 35 Stirling Highway, Crawley, WA 6009 Australia; 3 School of Physiotherapy and Exercise Science, Curtin University, GPO Box U1987, Perth, WA 6845 Australia

**Keywords:** Rett syndrome, Breathing disorders, Genotype, Rare disorder, International database, MECP2, Developmental disability

## Abstract

**Background:**

Rett syndrome is a severe neurodevelopmental disorder associated with mutations in the *MECP2* gene. Irregular breathing patterns and abdominal bloating are prominent but poorly understood features. Our aims were to characterize the abnormal breathing patterns and abdominal bloating, investigate the distribution of these by age and mutation type and examine their impact and management from a caregiver perspective.

**Methods:**

We invited previously recruited families from the International Rett Syndrome Study to complete a web-based questionnaire concerning their family member with Rett syndrome aged between 2 and 57 years. We used logistic regression to investigate presence, frequency and impact of breath-holding, hyperventilation, or abdominal bloating by age group and mutation type. Age of onset for both breathing abnormalities was investigated using time-to-onset analysis, and the Kaplan–Meier method was used to estimate the failure function for the study sample. Descriptive statistics were used to characterize the management of irregular breathing.

**Results:**

Questionnaires were returned by 413/482 (85.7%) families. Breath-holding was reported for 68.8%, hyperventilation for 46.4% and abdominal bloating for 42.4%. Hyperventilation was more prevalent and frequent in those younger than 7 years of age and abdominal bloating in those aged over 20 years. Onset of breathing irregularities usually occurred during early childhood. Caregivers perceived that daily life was considerably impacted for almost half (44.1%) of those with abdominal bloating and in just over than a third of those with breath-holding (35.8%) or hyperventilation (35.1%). Although perceived impact was broadly comparable between age and mutation groups for breath-holding, hyperventilation and abdominal bloating, girls and women with a p.Arg294* mutation were considered to be more affected by all three conditions. Only 31 individuals had received medically prescribed treatments including 12 different medications, added oxygen, rebreathing apparatus or non-invasive ventilation.

**Conclusions:**

Autonomic disturbances are prevalent and burdensome in Rett syndrome. This information may guide the design of inclusion criteria and outcome measures for clinical intervention trials targeting autonomic abnormalities. Further investigation of available treatments is necessary to delineate evidence-based management pathways.

## Background

Rett syndrome is a severe X-linked neurodevelopmental disorder affecting 1 in 9000 females and is generally associated with a loss-of-function mutation in the methyl CpG binding protein 2 gene (*MECP2*) [1–3]. Affected girls were typically considered to have an initial period of seemingly normal early development, though there is growing evidence to suggest that in a proportion, early development is also compromised [4, 5]. This apparently normal development is interrupted by a regression of acquired gross motor and communication skills and hand function between 6 and 18 months of age [1, 6]. Individuals commonly develop stereotypic hand movements and, if ambulant, an abnormal gait thereafter. Many will experience other severe comorbidities, including epilepsy, curvature of the spine, sleep disturbances, growth retardation, abnormal gastrointestinal issues and respiratory and autonomic dysfunction at variable ages of onset [1, 6, 7].

Approximately three quarters of girls and women with Rett syndrome survive into adulthood [7], and there is an imperative for better management of symptoms and illness that decrease quality of life. Irregular breathing patterns, generally ascribed to autonomic dysfunction in Rett syndrome, are a common and debilitating feature of the disease [6–9]. Types of breathing disturbances described in the literature include hyperventilation, breath-holding, central and obstructive apnoea, hypoventilation, apneustic breathing, Valsalva manoeuvres, tachypnoea and periodic breathing [8–10]. Breath-holding or apnoea, hyperventilation and interchange between these two abnormal respiratory rhythms are the most commonly described [8, 9, 11–15]. The exact pathways between loss of MeCP2 function and erratic patterns of breathing have yet to be eluciadated. Animal studies manipulating the expression of MeCP2 or its downstream products in different regions of the brain, related to respiratory function and modulation, indicate that multiple neural disturbances may combine to produce the differential breathing phenotypes demonstrated in patients with Rett syndrome [16–19]. Neurochemical imbalances and altered neuromodulation of synaptic interactions have been observed in multiple neurotransmitter systems, including the GABAergic and monoaminergic systems [16–19]. Oxidative stress [20, 21] and compensatory mechanisms for disturbed regulation of breathing are also thought to interfere with respiratory regulation and contribute to the variation of Rett syndrome breathing phenotypes described in the literature [17].

Although progress continues to be made towards understanding the mechanisms underlying the abnormal respiratory patterns [8, 18, 21–25], estimates of prevalence for these features have only been reported in relatively small samples (*n* < 150) and range widely between 58 and 94% for breath-holding [7, 10–12, 26–28] and 26 and 100% for hyperventilation [7, 10–12, 26–28]. Breath-holding and hyperventilation have been reported to occur in girls 7 years of age and younger (*n* = 47 [9], *n* = 12 [29]). However, information regarding the onset of irregular breathing has only been examined in a small case series [11] conducted prior to the discovery of the *MECP2* gene as the cause of Rett syndrome. The impacts of breath-holding or hyperventilation on affected individuals and their families have never been investigated. Several drug treatments that may potentially ameliorate irregular breathing have begun entering clinical trials [30], but presently, symptoms of irregular breathing are poorly controlled with few available treatments.

Abdominal bloating is thought to be related to the autonomic dysfunction and associated air swallowing in Rett syndrome [31, 32]. It is reported to affect between 50 and 60% of individuals with Rett syndrome [7, 27, 31]. Although abdominal bloating is believed to be associated with pain and discomfort [32], these symptoms do not appear to be consistently reported by caregivers [32].

Collecting new data from previously recruited families in the International Rett Syndrome Phenotype Database (InterRett) [33], our study aimed to characterize breath-holding, hyperventilation and abdominal bloating in Rett syndrome. We investigated the age of onset of breath-holding and hyperventilation, and the prevalence of each of these features by current age and mutation type. Additionally, we examined the impacts of these conditions on daily life as perceived by the family members and carers of individuals with Rett syndrome. We also described what treatments or strategies are currently being used to treat irregular respiratory patterns and their perceived effectiveness.

## Methods

### Data source

English-speaking families with a family member with a confirmed clinical diagnosis of Rett syndrome [6] and a pathogenic *MECP2* mutation who were part of the InterRett study [33] were invited to participate in the current study. A web-based questionnaire was developed principally to investigate respiratory and sleeping issues in Rett syndrome from parental perspectives and experiences, not usually described in clinical records. The questionnaire was organized as a series of short modules providing opportunity for informed consent and collecting information on current health and wellbeing of the individual with Rett syndrome, autonomic function, feeding difficulties and history of respiratory infections. It was made available to families using the software program REDCap (Research Electronic Data Capture, https://projectredcap.org). A paper format or telephone interview was also available on request. Ethics approval for this study was provided by The University of Western Australia Human Research Ethics Committee (RA/4/1/7449). The total number of responses used for analyses were stated in each case where the number differed from the entire sample due to incomplete or missing data.

### Sample characteristics

Country of residence, gender and date of birth of the individual with Rett syndrome were ascertained from each family during initial registration with InterRett. From our new questionnaire, the respondents were asked to identify their relationship to the individual with Rett syndrome from a list that included different familial relations and being a carer for the individual. These relationships were collapsed down to “natural parent”, “foster parent”, and “other family members”. Residential status was categorized as “parental home(s)”, “group home or community residential unit”, “hospital or nursing home”, or “other”. The respondents were asked to report whether the individual with Rett syndrome had ever been diagnosed with epilepsy or scoliosis, and seizures were classified as “not controlled (daily/more than once a week)”, “frequent (once per week)”, “occasional (once per month)”, “rare (once or twice per year)”, or “completely under control”.

### Covariates

Mutation types were grouped as C-terminal deletion, early truncating, large deletion, p.Arg106Trp, p.Arg133Cys, p.Arg168*, p.Arg255*, p.Arg270*, p.Arg294*, p.Arg306Cys and p.Thr158Met, and all other pathogenic mutations were grouped as “other”. Current age was categorized as “<7 years”, “7–12 years”, “13–19 years” or “≥20 years”. Mobility was grouped as “unable to walk”, “walks with assistance from a person or aid” or “walks with no assistance”.

### Outcomes

The respondents were asked to select the frequency at which episodes of breath-holding or hyperventilation were observed over the prior 3 months, and, if present, age of onset. Responses were categorized as “not at all”, “less than every day” and “every day or many times a day”. When breath-holding or hyperventilation was reported, the respondents were asked to describe their impact on the individual’s life by choosing from “None”, “Minor”, “Moderate” and “Major”. With the exception of questions about frequency, similar questions were also posed in relation to abdominal bloating. The respondents were also asked if the individual with Rett syndrome had ever been treated for irregular breathing. Those who answered in the affirmative were asked to describe the treatment and to rate its effectiveness as either “Worsened”, “No effect” or “Improved”.

### Statistical analysis

Descriptive statistics were used to summarize the characteristics of the affected individuals and to characterize breath-holding, hyperventilation, abdominal bloating and the management of irregular breathing. Logistic regression methods were employed to estimate the associations of mutation type and age with the categorical outcomes: presence and impact of the different breathing irregularities or abdominal bloating. For the frequency of irregular breathing, multinomial logistic regression was used to model the ordinal outcome and model associations of ordinal outcomes with mutation type and age. Analyses for age of onset of breath-holding and hyperventilation were restricted to a subset of responses describing affected individuals younger than 10 years of age to reduce the possibility of recall error. Age of onset was examined using time-to-onset analysis, and the Kaplan–Meier method was used to estimate the failure function for the study sample. The log-rank test was performed to evaluate the differences in age of onset by mutation type for both breath-holding and hyperventilation. All data were analysed using the statistical software program Stata 14 (Stata-Corp, College Station, TX).

## Results

### Sample characteristics

Questionnaires were returned by 413/482 (85.7%) families. The majority (81.7%) of families lived in the USA, and all but two cases (0.5%) were female. Nearly all (97.3%) respondents were the natural parents of the individual with Rett syndrome, and in most (93.7%) cases, the individual with Rett syndrome resided in the parental home. The frequency distributions of place of residence, age group, mutation type, current mobility, scoliosis and seizure diagnoses and current seizure frequency are shown in Table 1. The median age of the individuals with Rett syndrome was 14.5 years (first quartile 10.0 years, third quartile 20.9 years, range 2–57 years) with 12.4% under 7 years, just over a quarter (25.9%) aged at or over 20 years and the majority between 7 and 19 years. Nearly two fifths (39.2%) were able to walk independently, and 32% were unable to walk. Epilepsy and scoliosis had each been diagnosed in 68.4% of the sample. Of those diagnosed with epilepsy, 40.9% had seizures at least once a week at the time of data collection, while in just over a fifth (22.9%), the seizures were reported to be under control.

**Table 1:** Characteristics of the 413 individuals with Rett syndrome

Variables		*n* (%)
Gender	Female	411 (99.5)
	Male	2 (0.5)
Respondent	Natural parents	402 (97.3)
	Foster parents	6 (1.5)
	Other family members	5 (1.2)
Place of residence (*n* = 412)	Parental home(s)	386 (93.7)
	Group home or community residential unit	20 (4.9)
	Hospital or nursing home	1 (0.2)
	Other	5 (1.2)
Country of residence	USA	337 (81.6)
	Canada	32 (7.8)
	UK	29 (7.0)
	Australia	11(2.7)
	China	2 (0.5)
	Ireland	1 (0.2)
	Switzerland	1 (0.2)
Age distribution	0–6 years	51 (12.4)
	7–12 years	133 (32.2)
	13–19 years	122 (29.6)
	20 years and over	107 (25.9)
Mutation type	C-terminal deletion	44 (10.7)
	Early truncating	28 (6.8)
	Large deletion	28 (6.8)
	p.Arg106Trp	16 (3.9)
	p.Arg133Cys	30 (7.3)
	p.Arg168*	45 (10.9)
	p.Arg255*	46 (11.1)
	p.Arg270*	25 (6.1)
	p.Arg294*	30 (7.3)
	p.Arg306Cys	27 (6.5)
	p.Thr158Met	44 (10.7)
	Other	50 (12.1)
Current walking ability	Unable to walk	132 (32.0)
	Walks with assistance	119 (28.8)
	Walks with no assistance	162 (39.2)
Diagnosed with epilepsy (*n* = 411)	Yes	281 (68.4)
	No	130 (31.6)
Current seizure frequency (*n* = 279)	Not controlled (daily/more than once a week)	53 (19.0)
	Frequent (once per week)	61 (21.9)
	Occasional (once per month)	60 (21.5)
	Rare (once or twice per year)	41 (14.7)
	Completely under control	64 (22.9)
Diagnosed with scoliosis (*n* = 409)	Yes	280 (68.5)
	No	129 (31.5)

### Age of onset for observed breathing irregularities

**Fig. 1:**
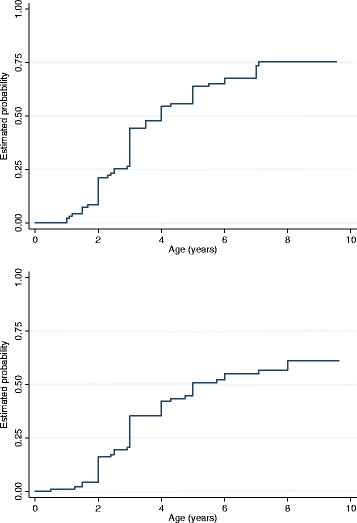
Kaplan–Meier failure functions for age of onset of autonomic disturbances in girls and women with Rett syndrome who were younger than 10 years old at time of ascertainment. *Top*: breath-holding (*n* = 95). *Bottom*: hyperventilation (*n* = 93)

Time-to-onset analysis showed that by the age of 5 years, breath-holding would have been reported for 63.8% (95% confidence interval [CI] 53.9, 73.7%) and hyperventilation for 50.8% (95% CI 40.8, 61.7%) (Fig. 1). Mutation type did not influence the age of onset of breath-holding (log-rank test *χ* ^2^ (11) = 14.60, *p* = 0.202) or hyperventilation (log-rank test *χ* ^2^ (11) = 9.15, *p* = 0.608).

### Prevalence of current autonomic disturbances

In the 3 months prior to data collection, 74% (281/380) of the girls and women with Rett syndrome were reported to have had irregular breathing patterns. Episodes of breath-holding were more commonly reported (68.8%, 260/378) than episodes of hyperventilation (46.4%, 179/386). Abdominal bloating was observed in 42.4% (173/408) (Table 2). Compared to the youngest age group (≤7 years), the odds of hyperventilation were lower for those who were 7 years and older, after accounting for mutation type (Table 3). In contrast, the odds of experiencing abdominal bloating were more than 70% higher for the oldest age group (20 years and older) than for the youngest one, irrespective of mutation type. Associations between mutation types and breathing irregularities and abdominal bloating were not evident in the study sample (Table 3).

**Table 2:** Prevalence of current autonomic disturbances by age group and mutation type

	Breath-holding	Hyperventilation	Both breath-holding and hyperventilation	Abdominal bloating
	*n*/*N* (%)	*n*/*N* (%)	*n*/*N* (%)	*n*/*N* (%)
Overall	260/378 (68.8)	179/386 (46.4)	158/384 (41.2)	173/408 (42.4)
Age group				
0–6 years	32/49 (65.3)	29/48 (60.4)	27/48 (56.3)	20/51 (39.2)
7–12 years	99/130 (76.2)	59/130 (45.4)	59/130 (45.4)	48/131 (36.6)
13–19 years	76/113 (67.3)	48/114 (42.1)	38/114 (33.3)	49/121 (40.5)
20 years and above	53/86 (61.6)	43/94 (45.7)	34/92 (37.0)	56/105 (53.3)
Mutation type				
C-terminal deletion	28/41 (68.3)	17/42 (40.5)	14/42 (33.3)	16/44 (36.4)
Early truncating	23/28 (82.1)	13/27 (48.2)	13/27 (48.2)	14/28 (50.0)
Large deletion	21/26 (80.8)	12/26 (46.2)	12/26 (46.2)	12/28 (42.9)
p.Arg106Trp	10/15 (66.7)	7/15 (46.7)	4/15 (26.7)	6/15 (40.0)
p.Arg133Cys	19/29 (65.5)	15/30 (50.0)	13/30 (43.3)	12/30 (40.0)
p.Arg168*	26/40 (65.0)	19/43 (44.2)	16/42 (38.1)	22/44 (50.0)
p.Arg255*	32/44 (72.7)	22/44 (50.0)	21/44 (47.7)	18/46 (39.1)
p.Arg270*	17/24 (70.8)	11/24 (45.8)	10/24 (41.7)	10/25 (40.0)
p.Arg294*	18/27 (66.7)	12/27 (44.4)	11/27 (40.7)	10/29 (34.5)
p.Arg306Cys	18/26 (69.2)	15/26 (57.7)	13/25 (52.0)	11/27 (40.7)
p.Thr158Met	24/34 (70.6)	18/36 (50.0)	17/36 (47.2)	23/43 (53.5)
Other	24/44 (54.6)	18/46 (39.1)	14/46 (30.4)	19/49 (38.4)

**Table 3:** Logistic regression analysis of the associations between breathing irregularities, age and mutation type

	Breath-holding (*n* = 378)				Hyperventilation (*n* = 386)				Abdominal bloating (*n* = 408)			
	Crude OR (95% CI)	*p*	Adjusted OR (95% CI)	*p*	Crude OR (95% CI)	*p*	Adjusted OR (95% CI)	*p*	Crude OR (95% CI)	*p*	Adjusted OR (95% CI)	*p*
Age group												
0–6	REF		REF		REF		REF		REF		REF	
7–12	1.70 (0.83, 3.46)	0.15	1.53 (0.73, 3.20)	0.26	0.54 (0.28, 1.07)	0.08	0.49 (0.25, 0.99)	0.05	0.90 (0.46, 1.74)	0.75	0.88 (0.45, 1.75)	0.72
13–19	1.09 (0.54, 2.21)	0.81	1.02 (0.49, 2.11)	0.96	0.48 (0.24, 0.95)	0.04	0.45 (0.22, 0.92)	0.03	1.05 (0.54, 2.06)	0.88	1.08 (0.54, 2.14)	0.83
20+	0.85 (0.41, 1.77)	0.67	0.80 (0.38, 1.69)	0.56	0.55 (0.27, 1.12)	0.10	0.54 (0.26, 1.10)	0.09	1.77 (0.90, 3.50)	0.10	1.79 (0.90, 3.58)	0.10
Mutation type												
C-terminal deletion	REF		REF		REF		REF		REF		REF	
Early truncating	2.14 (0.66, 6.88)	0.20	2.21 (0.68, 7.16)	0.19	1.37 (0.52, 3.62)	0.53	1.38 (0.52, 3.69)	0.52	1.75 (0.67, 4.58)	0.25	1.78 (0.67, 4.71)	0.24
Large deletion	1.95 (0.60, 6.32)	0.27	1.80 (0.55, 5.89)	0.33	1.26 (0.47, 3.38)	0.65	1.27 (0.47, 3.43)	0.64	1.31 (0.50, 3.46)	0.58	1.47 (0.55, 3.92)	0.44
p.Arg106Trp	0.93 (0.26, 3.27)	0.91	1.05 (0.29, 3.72)	0.95	1.29 (0.39, 4.22)	0.68	1.28 (0.39, 4.23)	0.69	1.17 (0.35, 3.88)	0.80	1.04 (0.31, 3.53)	0.94
p.Arg133Cys	0.88 (0.32, 2.42)	0.81	0.84 (0.30, 2.32)	0.73	1.47 (0.57, 3.78)	0.42	1.52 (0.59, 3.92)	0.39	1.17 (0.45, 3.03)	0.75	1.22 (0.47, 3.20)	0.69
p.Arg168*	0.86 (0.34, 2.17)	0.75	0.91 (0.36, 2.33)	0.85	1.16 (0.49, 2.75)	0.73	1.04 (0.43, 2.49)	0.93	1.75 (0.75, 4.10)	0.20	1.67 (0.70, 3.97)	0.25
p.Arg255*	1.24 (0.49, 3.15)	0.65	1.27 (0.50, 3.27)	0.62	1.47 (0.63, 3.45)	0.38	1.41 (0.60, 3.34)	0.43	1.13 (0.48, 2.64)	0.79	1.13 (0.48, 2.67)	0.79
p.Arg270*	1.13 (0.38, 3.38)	0.83	1.22 (0.40, 3.73)	0.72	1.24 (0.45, 3.42)	0.67	1.14 (0.41, 3.17)	0.80	1.17 (0.43, 3.20)	0.77	1.09 (0.39, 3.03)	0.87
p.Arg294*	0.93 (0.33, 2.62)	0.89	0.96 (0.34, 2.73)	0.94	1.18 (0.44, 3.12)	0.75	1.18 (0.44, 3.15)	0.74	0.92 (0.35, 2.46)	0.87	0.88 (0.33, 2.39)	0.81
p.Arg306Cys	1.04 (0.36, 3.02)	0.94	0.99 (0.34, 2.89)	0.99	2.01 (0.74, 5.41)	0.17	1.91 (0.70, 5.20)	0.21	1.20 (0.45, 3.22)	0.71	1.30 (0.48, 3.51)	0.60
p.Thr158Met	1.11 (0.41, 2.99)	0.83	1.13 (0.42, 3.05)	0.82	1.47 (0.60, 3.61)	0.40	1.43 (0.58, 3.52)	0.44	2.01 (0.85, 4.75)	0.11	1.97 (0.83, 4.69)	0.13
Other mutations	0.56 (0.23, 1.35)	0.20	0.60 (0.25, 1.48)	0.27	0.94 (0.40, 2.22)	0.90	0.85 (0.36, 2.04)	0.72	1.11 (0.48, 2.57)	0.81	1.07 (0.46, 2.52)	0.87

OR was adjusted for age group and mutation type

*OR* odds ratio, *CI* confidence interval, *REF* reference category

### Relationship between current autonomic disturbances

Hyperventilation was reported for just over three fifths (62.7%, 158/252) of individuals who experienced breath-holding. Just under half (48.5%, 125/258) of those with breath-holding experienced abdominal bloating, as did 57.1% (101/177) of those with hyperventilation. Accounting for age and mutation type, breath-holding and hyperventilation were each associated with increased odds of having abdominal bloating (breath-holding: odds ratio [OR] 3.06, 95% CI 1.85,5.06; hyperventilation: OR 3.72, 95% CI 2.39,5.78).

### Frequency of breathing irregularities

Breath-holding occurred every day or many times a day in slightly fewer than half (47.9%, 181/378), whereas only just over a quarter (26.4%, 102/386) experienced frequent hyperventilation (Table 4). Frequent breath-holding was observed for just over a half (52.4%, 153/292) of those under 20 years, but only for one third (32.6%, 28/86) of those 20 years and older. Frequent hyperventilation also declined with age (Table 4). After accounting for mutation type, the relative risk ratios of frequent hyperventilation compared to none at all were lower in those aged 7 years and above than those in the youngest age group (Table 5). The relative risk ratios of frequent and less frequent breathing irregularities were comparable among mutation types (Table 5).

**Table 4:** Frequency of breathing irregularities by age group and mutation type

	Breath-holding				Hyperventilation			
	Not at all	Less than everyday	Every day or many times a day	Total	Not at all	Less than everyday	Every day or many times a day	Total
*n* (%^a^)	118 (31.2)	79 (20.9)	181 (47.9)	378	207 (53.6)	77 (20.0)	102 (26.4)	386
Age group, *n* (%^a^)								
0–6 years	17 (34.7)	8 (16.3)	24 (49.0)	49	19 (39.6)	5 (10.4)	24 (50.0)	48
7–12 years	31 (23.9)	30 (23.1)	69 (53.1)	130	71 (54.6)	19 (14.6)	40 (30.8)	130
13–19 years	37 (32.7)	16 (14.2)	60 (53.1)	113	66 (57.9)	26 (22.8)	22 (19.3)	114
20 years and above	33 (38.4)	25 (29.1)	28 (32.6)	86	51 (54.3)	27 (28.7)	16 (17.0)	94
Mutation type, *n* (%^a^)								
C-terminal deletion	13 (31.7)	7 (17.1)	21 (51.2)	41	25 (59.5)	4 (9.5)	13 (31.0)	42
Early truncating	5 (17.9)	6 (21.4)	17 (60.7)	28	14 (51.9)	4 (14.8)	9 (33.3)	27
Large deletion	5 (19.2)	7 (26.9)	14 (53.9)	26	14 (53.8)	6 (23.1)	6 (23.1)	26
p.Arg106Trp	5 (33.3)	5 (33.3)	5 (33.3)	15	8 (53.3)	4 (26.7)	3 (20.0)	15
p.Arg133Cys	10 (34.5)	6 (20.7)	13 (44.8)	29	15 (50.0)	7 (23.3)	8 (26.7)	30
p.Arg168*	14 (35.0)	10 (25.0)	16 (40.0)	40	24 (55.8)	11 (25.6)	8 (18.6)	43
p.Arg255*	12 (27.3)	5 (11.4)	27 (61.4)	44	22 (50.0)	7 (15.9)	15 (34.1)	44
p.Arg270*	7 (29.2)	7 (29.2)	10 (41.6)	24	13 (54.2)	4 (16.6)	7 (29.2)	24
p.Arg294*	9 (33.3)	5 (18.5)	13 (48.2)	27	15 (55.6)	2 (7.4)	10 (37.0)	27
p.Arg306Cys	8 (30.8)	5 (19.2)	13 (50.0)	26	11 (42.3)	5 (19.2)	10 (38.5)	26
p.Thr158Met	10 (29.4)	7 (20.6)	17 (50.0)	34	18 (50.0)	9 (25.0)	9 (25.0)	36
Other	20 (45.5)	9 (20.5)	15 (34.1)	44	28 (60.9)	14 (30.4)	4 (8.7)	46

^a^Row percentage

**Table 5:** Multinomial logistic regression analysis of the associations between frequency of breathing irregularities and age and mutation type

	Breath-holding (*n* = 378)				Hyperventilation (*n* = 386)			
	Less than every day		Every day or many times a day		Less than every day		Every day or many times a day	
	Adjusted RRR (95% CI)	*p*	Adjusted RRR (95% CI)	*p*	Adjusted RRR (95% CI)	*p*	Adjusted RRR (95% CI)	*p*
Age group								
0–6 years	REF		REF		REF		REF	
7–12 years	1.94 (0.71, 5.30)	0.20	1.39 (0.63, 3.03)	0.41	1.13 (0.36, 3.54)	0.83	0.32 (0.15, 0.69)	<0.01
13–19 years	0.90 (0.31, 2.55)	0.84	1.05 (0.49, 2.28)	0.90	1.74 (0.57, 5.26)	0.33	0.19 (0.08, 0.45)	<0.01
20 years and above	1.51 (0.55, 4.13)	0.42	0.56 (0.25, 1.27)	0.17	2.33 (0.77, 7.08)	0.14	0.19 (0.08, 0.46)	<0.01
Mutation type								
C-terminal deletion	REF		REF		REF		REF	
Early truncating	2.35 (0.52, 10.65)	0.27	2.15 (0.63, 7.33)	0.22	1.79 (0.38, 8.37)	0.46	1.26 (0.42, 3.79)	0.68
Large deletion	2.49 (0.57, 10.96)	0.23	1.57 (0.45, 5.45)	0.48	3.02 (0.72, 12.69)	0.13	0.74 (0.22, 2.47)	0.63
p.Arg106Trp	1.96 (0.41, 9.33)	0.40	0.71 (0.17, 2.99)	0.64	2.82 (0.56, 14.10)	0.21	0.77 (0.17, 3.52)	0.73
p.Arg133Cys	1.07 (0.27, 4.25)	0.92	0.76 (0.26, 2.25)	0.62	3.07 (0.76, 12.38)	0.12	1.04 (0.34, 3.15)	0.95
p.Arg168*	1.35 (0.39, 4.67)	0.64	0.76 (0.27, 2.09)	0.59	2.90 (0.80, 10.51)	0.11	0.47 (0.16, 1.41)	0.18
p.Arg255*	0.81 (0.20, 3.28)	0.77	1.43 (0.53, 3.81)	0.48	2.00 (0.51, 7.80)	0.32	1.20 (0.46, 3.16)	0.71
p.Arg270*	1.85 (0.45, 7.54)	0.39	0.99 (0.30, 3.33)	0.99	1.84 (0.39, 8.68)	0.44	0.89 (0.27, 2.90)	0.84
p.Arg294*	1.10 (0.26, 4.64)	0.90	0.91 (0.30, 2.77)	0.87	0.780 (0.13, 4.92)	0.81	1.35 (0.46, 3.94)	0.58
p.Arg306Cys	1.16 (0.27, 4.98)	0.85	0.93 (0.30, 2.88)	0.90	3.15 (0.70, 14.18)	0.14	1.46 (0.47, 4.48)	0.51
p.Thr158Met	1.30 (0.34, 4.95)	0.70	1.07 (0.37, 3.06)	0.90	3.23 (0.85, 12.27)	0.08	0.86 (0.29, 2.51)	0.78
Other mutations	0.96 (0.28, 3.26)	0.94	0.49 (0.18, 1.30)	0.15	3.20 (0.92, 11.18)	0.07	0.19 (0.05, 0.71)	0.01

The base outcome for the analyses was “Not at all”. RRR was adjusted for age group and mutation type

*RRR* relative risk ratio, *CI* confidence interval, *REF* reference category

### Impact of observed autonomic disturbances on daily life

Abdominal bloating was considered to have a moderate or major impact on the individual’s life in nearly half (44.1%, 75/170) of those reported to experience the condition. As one caregiver described,

It is causing pseudo bowel obstructions and she had to have a g-tube and cecostomy placed because of the air swallowing due to the breath-holding. When she holds her breath her tummy can become so distended that she looks like she is pregnant and she needs to be vented of the air throughout her stomach and intestines.

Breath-holding and hyperventilation were less commonly reported than abdominal bloating to have a moderate or major impact on an individual’s life (35.8%, 92/257 and 35.1%, 60/171, respectively). These can impact on daily functioning and focus, for example:

She has difficulty concentrating and focusing on tasks. It severely impacts her eating.

She gets very tense and unstable if she is standing. Sometimes I think she is getting light headed.

**Fig. 2:**
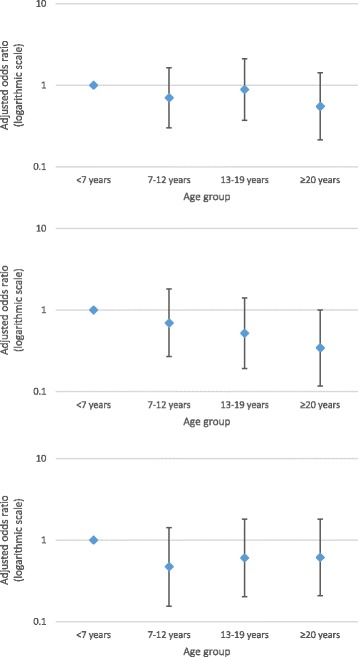
Relationship of reporting moderate or major impact on daily life to age groups for those with breath-holding (*top*, *n* = 257), hyperventilation (*middle*, *n* = 171) and abdominal bloating (*bottom*, *n* = 170), adjusted for mutation type

**Fig. 3:**
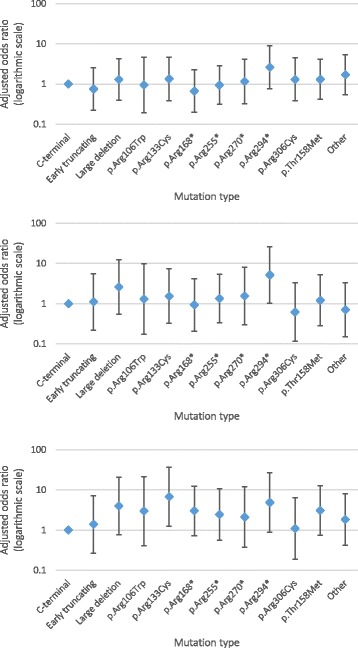
Relationship of reporting moderate or major impact on daily life to common mutation type for those with breath-holding (*top*, *n* = 257), hyperventilation (*middle*, *n* = 171) and abdominal bloating (bottom, *n* = 170), adjusted for age

Girls and women with a p.Arg294* mutation had the highest perceived impact for both breath-holding (55.6%, 10/18) and hyperventilation (66.7%, 8/12) and were second to those with a p.Arg133Cys mutation (60%, 6/10 and 66.7%, 8/12, respectively) for moderate or major impact from abdominal bloating. The perceived impacts for observed autonomic disturbances were broadly similar between age groups and mutation types (Figs. 2 and 3). After accounting for age, girls and women with a p.Arg294* mutation had greater odds of being perceived as experiencing a moderate or major impact from hyperventilation (OR = 5.18, 95% CI 1.03,26.07) and abdominal bloating (OR = 4.86, 95% CI 0.88,26.83). Those with a p.Arg133Cys mutation also had greater odds (OR = 6.77, 95% CI 1.26,36.29) of being perceived as experiencing a moderate or major impact from abdominal bloating.

### Management of irregular breathing

Thirty-one individuals received a medically prescribed treatment for their breathing irregularities, as shown with their perceived effects in Table 6. Caregivers observed improvement for all four individuals prescribed topiramate and for the two individuals prescribed acetazolamide, whereas improvement following use of buspirone was mixed. Breathing irregularities were perceived to be improved in all who were prescribed CPAP/BIPAP and in two thirds who were prescribed rebreathing techniques or added oxygen. A preparation containing magnesium had been prescribed for 21 individuals to manage constipation, and only one of these was experiencing a major impact from breathing irregularities suggesting that it might be providing a beneficial effect on these.

**Table 6:** Frequency of medically prescribed treatments for irregular breathing (*n* = 31)

Treatment type	Perceived effect of treatment		Total
	Not improved	Improved	
Medications	8	13	21
Antiepilepsy drugs^a^	–	5	5
Serotonin agonist^b^	3	3	6
Benzodiazepine^c^	1	–	1
SSRIs^d^	5	1	6
Other^e^	3	4	7
Use of equipment^f^	4	11	15
Oxygen	2	4	6
Rebreathing apparatus	1	2	3
CPAP/BIPAP	–	5	5
Compression vest	1	–	1

Included sole and combined usage of the medications/equipment

^a^Topiramate (*n* = 4) and carbamazepine (*n* = 1)

^b^Buspirone

^c^Diazepine

^d^Citalopram, escitalopram, fluoxetine, sertraline

^e^Naltrexone, propranolol, acetazolamide, clonidine, both individuals taking acetazolamide were perceived to improve

^f^Equipment included use of any added gases or apparatus

## Discussion

Breath-holding was reported by caregivers for approximately two thirds of the sample, whereas just under half were said to experience hyperventilation or abdominal bloating. Onset usually occurred during early childhood. The prevalence of irregular breathing and abdominal bloating were comparable between mutation groups, but hyperventilation and abdominal bloating were affected differently by age. Frequent breath-holding and hyperventilation became less evident while abdominal bloating became more evident with increasing age. More than four fifths of those with hyperventilation (88.3%) and those with abdominal bloating (81.7%) also experienced breath-holding. Approximately one quarter of the sample were reported to experience all three conditions. The impacts of breath-holding, hyperventilation and abdominal bloating were perceived as broadly similar across age and mutation groups, although those individuals with a p.Arg294* mutation were considered to be more impacted by all three conditions.

These prevalence data are consistent with proportions reported by smaller sample size (*n* = 53 [26], *n* = 143 [34], *n* = 145 [35]) parent-report studies for breath-holding. Slightly higher prevalences of breath-holding (76.9%) and hyperventilation (58.2%) were reported in a smaller (*n* = 91) and slightly older (4–47 years, mean 20.5 years) Italian population [28]. Conversely, parent-report studies with older (≥16 years, *n* = 53) [27] or exclusively adult populations (>18 years, *n* = 146) [7] reported lower prevalence of hyperventilation (49.3 and 39%, respectively) in their samples. The higher prevalence of abdominal bloating we found in older individuals was consistent with other studies with older populations [7, 27].

Direct measurement of respiratory function in individuals with Rett syndrome has suggested that all subjects experience breath-holding [9, 10, 15, 29] and hyperventilation [15, 29]. This differential between parent-report studies and clinical studies may in part result from the small sample size of the clinical studies (*n* < 48) [9, 10, 15, 29] or because some breathing irregularities are very subtle and thus not observable by family members or carers. By contrast, the proportion of patients with Rett syndrome reported with abdominal bloating in a small clinical study (*n* = 33, 61%) [31] was broadly comparable to the prevalence found in this parent-report study (42.2%). Ours is the largest genetically confirmed sample to compare the individual prevalences of breath-holding, hyperventilation and abdominal bloating across all age groups, giving strength to the findings.

We found that autonomic disturbances were first observed during early childhood for many individuals with earlier onset of breath-holding (occurring in half by age of 4 years) compared with hyperventilation (occurring in a half by age of 5 years). The mean age of regression in Rett syndrome has been reported as 19.3 months [36], and the median ages of loss of speech and hand function at 18 and 22 months, respectively [37]. Thus, in comparison, the onsets of breath-holding and hyperventilation, we identified, are occurring somewhat later, generally after the regression period. As with other clinical features [38], the age of regression also varies by genotype and is, for example, 10 months earlier in those with p.Arg106Trp than in those with C-terminal deletions [36]. Therefore, it is not possible to specify exactly the relationship between the stage of developmental regression as defined by Hagberg and Witt Engerstrom [39] 1986 and the onset of breathing irregularities. However, despite the known demonstrated effect of genotype on regression age [36], we did not find in this analysis that mutation type influenced the age of onset reported for autonomic disturbances, possibly because of the truncated sample used for the time-to-event analyses.

Daily episodes of breath-holding and hyperventilation were reported for almost a half and just over a quarter of the sample, respectively. Although we did not identify any relationship with mutation type, frequent hyperventilation declined with age. This may suggest that maturation leads to a real reduction in frequency of hyperventilation. Data from animal studies investigating potential mechanisms for respiratory irregularities suggest that respiratory control may be disturbed by a number of altered and interacting neuronal pathways that change with developmental age [17, 19]. These may contribute to multiple breathing rhythms being displayed by one individual over a lifetime [17, 29] and could potentially contribute to less frequent observation of irregular breathing with age as seen in our data. However, it is also possible that parents of older individuals may have become more familiar with daily episodes of hyperventilation or breath-holding and were therefore less likely to report their presence. Alternatively, these findings might result from a survival bias, whereby individuals with more severe and frequent irregular breathing have died. The latter hypothesis is supported by the parent-reported decline in breath-holding spells and hyperventilation in a 5-year longitudinal study of Dutch women with Rett syndrome [27]. Initially 73 and 39% of the cohort were reported to have breath-holding spells and hyperventilation, respectively. Five years later, seven women had died, and 60 and 26% of the smaller cohort (*n* = 37) were reported to experience breath-holding and hyperventilation, supporting the survival bias hypothesis. However, further longitudinal research that encompasses younger individuals with Rett syndrome is required to confirm these findings and the underlying mechanisms.

The presence of one autonomic disturbance was associated with an increased risk of having either of the other two autonomic disturbances, with the strongest of these relationships being the association between breath-holding and hyperventilation. This relationship is consistent with earlier studies where it was hypothesized that either hyperventilation induced breath-holding [12] or that breath-holding induced hyperventilation [40]. More recent physiological studies suggest that there is no single relationship between these two types of irregular breathing and that several irregular breathing patterns that include breath-holding and hyperventilation exist in Rett syndrome [8, 29]. One study measuring respiratory function in 12 subjects with Rett syndrome using remote cardio-respiratory event monitoring in the home environment found that no dominant pattern emerged, but each individual demonstrated their own unique dominant pattern that included shallow breathing, hypoventilation and/or apnoea [29]. We also found that abdominal bloating commonly co-occurred with both hyperventilation and breath-holding. Consistently, a study (*n* = 33) monitoring respiration and swallowing at rest determined that air swallowing could occur concurrently with both breath-holding and hyperventilation [31]. Of the 20 individuals with abdominal bloating, 17 swallowed air during breath-holding and the remaining three “gulped” air during hyperventilation. It is possible that breath-holding is more likely to result in swallowed air, whereas hyperventilation is not as likely to lead to swallowed air, but those who do concurrently hyperventilate and swallow air take in more air through “gulping” and are therefore at a greater risk of developing abdominal bloating.

Caregivers perceived considerable impacts on daily life for almost half of those with abdominal bloating and just over a third of those with breath-holding or hyperventilation. In a population-based study on outcomes following gastrostomy, 39 of 66 with a gastrostomy also experienced abdominal bloating and 55% of those caregivers observed reduced impact following gastrostomy [41]. Abdominal bloating can be extremely uncomfortable, and gastrostomy has also been recommended in individual cases for venting and relief of pain [31, 32, 41]. Girls and women with a p.Arg294* mutation were perceived to be substantially affected by autonomic disturbances more often than girls and women in any other mutation group. Serotonin neurotransmitters are important regulators of respiratory patterns and mood [25], and in an Australian population-based study, individuals with a p.Arg294* mutation were also more likely to experience mood disturbance in comparison to those with other mutations [35]. The mutation p.Arg294* is usually associated with a mild overall phenotype and less severe clinical features [42–45]. However, it is possible that the deficits of serotonergic neurotransmission in Rett syndrome [25, 46] are greater for individuals with a p.Arg294* mutation, and therefore, the regulation of breathing and mood in these individuals is more affected than those with other mutation types.

The minority of those with autonomic dysfunction and moderate to severe impact on daily life received a medically prescribed treatment, not surprising given that available literature is limited mainly to case studies [47–51] and two case series (*n* = 7 [52] and 8 [53]). Interestingly, six caregivers in our study observed mixed effects in relation to the use of buspirone in contrast to positive effects described in two case studies [47, 49], and only a minority of those prescribed SSRI medications appeared to respond favourably in contrast with the positive effect seen in one case study [49]. Two individuals in our sample had been prescribed naltrexone which has been found associated with adverse effects in a previous clinical trial [54]. The use of topiramate and acetazolamide were each associated with benefits for a small number of individuals in our study, consistent with observations for nine of 10 patients [51, 53] and one of two patients [51], respectively. A previous case series reported reduced autonomic breathing disturbances following prescribed magnesium citrate [52], but this had not been prescribed for autonomic disturbances in our sample although we did observe that those taking magnesium for constipation were not severely impacted by their breathing disturbances. Supported respiration with CPAP or BIPAP was useful for some [48], and no individual had been prescribed carbogen [50]. Further trials are desperately needed to delineate evidence-based management pathways, and our data suggests that topiramate and magnesium citrate could be worthy of further investigation.

Parents were asked to provide information regarding the onset of conditions that typically appear during early childhood. Length of recall and the related effects of memory lapse, whereby the ability to remember events declines over time, and telescoping, where past events are recalled as happening more recently [55], are therefore issues which have to be considered in studies such as ours. In such studies, increased salience of symptoms may influence recall positively while mild symptoms may be under-reported because of memory lapse for less significant events [56–58]. We acknowledge that recall for age at onset of breath-holding may be better than for hyperventilation because it is likely to be of more concern to parents. Our study therefore sought to balance the risk of recall bias with the need for an adequate sample size by asking parents targeted questions about the time period around the event and by limiting the time-to-event analyses to those under 10 years of age. However, our conclusions should still be tempered by the potential for some recall bias.

We acknowledge that our study has other limitations. Chance associations are possible due to the multiple comparisons made in our paper. We also recognize that these data are parent-reported and that differences from clinical estimates may arise from difficulties observing the potentially subtle nature of autonomic disturbances. Future research could undertake direct physiological assessment using a large representative sample and correlate these data with parent observation of autonomic abnormalities.

## Conclusions

This is the first study to investigate caregiver perspectives on breath-holding, hyperventilation and abdominal bloating in Rett syndrome using a large international sample. The wide range of ages in this genetically confirmed cohort brings strength to the estimated prevalence of these autonomic conditions. This is also the first time that the age of onset for irregular breathing and the perceived impacts of irregular breathing and abdominal bloating have been investigated including relationships with genotype.

Although the perceived impact of autonomic disturbances by caregivers may be considered subjective, parental perspectives are important for prioritizing where there is the greatest need for improving clinical management. Potential treatments for breath-holding and hyperventilation are already beginning to enter trials where parental perspectives are an important outcome [59, 60]. The autonomic data reported here may guide researchers in improving inclusion criteria and outcome measures for these clinical intervention trials, hopefully leading to the availability of better management strategies for autonomic conditions in individuals with Rett syndrome.

## Abbreviations

BIPAP: Bilevel positive airway pressure
CI: Confidence interval
CPAP: Continuous positive airway pressure
g-tube: Gastrostomy tube
InterRett: International Rett Syndrome Phenotype Database
MECP2: Methyl CpG binding protein 2
OR: Odds ratio
REDCap: Research Electronic Data Capture
